# Interdigitated aluminium and titanium sensors for assessing epithelial barrier functionality by electric cell-substrate impedance spectroscopy (ECIS)

**DOI:** 10.1007/s10544-020-00486-4

**Published:** 2020-04-24

**Authors:** Thomas Schmiedinger, Stefan Partel, Thomas Lechleitner, Oliver Eiter, Daniel Hekl, Stephan Kaseman, Peter Lukas, Johannes Edlinger, Judith Lechner, Thomas Seppi

**Affiliations:** 1 Laboratory of Radiobiology, University Hospital for Radiotherapy and Radiation Oncology, Medical University of Innsbruck, Anichstraße 35, A-6020 Innsbruck, Austria; 2 University of Applied Sciences Kufstein, Andreas Hofer-Straße 7, A-6330 Kufstein, Austria; 3 Research Centre for Microtechnology, Vorarlberg University of Applied Sciences, Hochschulstraße 1, A-6850 Dornbirn, Austria; 4 Department of Physiology and Medical Physics, Medical University of Innsbruck, Schöpfstraße 41/EG, A-6330 Innsbruck, Austria

**Keywords:** Electric cell-substrate impedance spectroscopy, Interdigitated electrode array, Epithelial cells, Equivalent electrical circuit

## Abstract

Electric cell-substrate impedance spectroscopy (ECIS) enables non-invasive and continuous read-out of electrical parameters of living tissue. The aim of the current study was to investigate the performance of interdigitated sensors with 50 μm electrode width and 50 μm inter-electrode distance made of gold, aluminium, and titanium for monitoring the barrier properties of epithelial cells in tissue culture. At first, the measurement performance of the photolithographic fabricated sensors was characterized by defined reference electrolytes. The sensors were used to monitor the electrical properties of two adherent epithelial barrier tissue models: renal proximal tubular *LLC-PK1* cells, representing a normal functional transporting epithelium, and human cervical cancer-derived *HeLa* cells, forming non-transporting cancerous epithelial tissue. Then, the impedance spectra obtained were analysed by numerically fitting the parameters of the two different models to the measured impedance spectrum. Aluminium sensors proved to be as sensitive and consistent in repeated online-recordings for continuous cell growth and differentiation monitoring as sensors made of gold, the standard electrode material. Titanium electrodes exhibited an elevated intrinsic ohmic resistance in comparison to gold reflecting its lower electric conductivity. Analysis of impedance spectra through applying models and numerical data fitting enabled the detailed investigation of the development and properties of a functional transporting epithelial tissue using either gold or aluminium sensors. The result of the data obtained, supports the consideration of aluminium and titanium sensor materials as potential alternatives to gold sensors for advanced application of ECIS spectroscopy.

## Introduction

The key function of epithelial tissue is to maintain homeostasis by creating and maintaining compartments with different solute composition (Zihni et al. 2016). Epithelial tissues create barriers and allow regulated selective site-directed transport of electrolytes, nutrients and waste products (Günzel 2017; Tanos and Rodriguez-Boulan 2008). Size- and charge-selective transport is possible across the epithelial barrier given the presence of conductive pores within the tight junctional complex achieved through the incorporation of specific cation- or anion-selective and water-channel forming transmembrane proteins of the claudin family (Zihni et al. 2016). The resulting ion permeability can be assessed experimentally by quantifying the trans-epithelial electrical resistance (TEER) of the epithelium. The TEER value represents the ohmic resistance between the apical and basolateral side and can be measured directly by placing electrodes on the upper and lower side of a cell layer grown on a permeable filter to provide growth support (Srinivasan et al. 2015). The ion permeation ability of an epithelium can also be assessed by electric cell–substrate impedance spectroscopy (ECIS), which is an impedance-based measurement method for analysing the complex electrical impedance of cells grown on top of planar electrodes (Günzel et al. 2012). The ECIS method was introduced as a novel method for studying cell-cell and cell-substrate interactions in real-time and non-invasively (Giaever and Keese 1984). The frequency-dependant impedance is determined by applying a sinusoidal voltage of low amplitude to the sensing element and measuring the response of the system (Lisdat and Schäfer 2008). In addition to the assessment of impedance at a single frequency, which has the advantage of enabling a higher temporal resolution of the measurements (Szulcek et al. 2014), impedance can be assessed at different frequencies resulting in an impedance spectrum, which allows the reconstruction and estimation of key physico-chemical parameters of the system under investigation (Bondarenko 2012). This reconstruction is done via a two-step approach: by (i) modelling the electrical behaviour of the system under investigation and (ii) fitting the parameters of the model to the measured impedance spectrum. In addition to applications of ECIS for the study of fundamental cellular processes such as attachment (Lo et al. 1999), proliferation (Wang et al. 2010), migration (Hong et al. 2011), differentiation (Bagnaninchi and Drummond 2011; Ertl et al. 2014), the assessment of tissue functionality of epithelial / endothelial barrier properties has also been reported (Wegener et al. 2000).

An essential part of all ECIS systems is the sensing electrode, whose uniqueness is based on the electrode material and the design of the measurement electrode. An established material for ECIS sensors is gold, which is known as an excellent biocompatible material (Wintermantel and Ha 2009). In addition, gold also includes outstanding electrical properties, which in combination with the biological response makes the material well suited as electrode material for biosensors. However, gold may not be the sole choice for the electrode material of ECIS biosensor. Other highly conductive and more cost-effective materials, like titanium or aluminium, may be suited for ECIS as well. Regarding biocompatibility of the aforementioned materials, titanium is a known material for medical body implants (Niinomi 1999; Wintermantel and Ha 2009). In the case of aluminium, a study by Eisenbarth et al. (2004) showed that the proliferation activity of cells grown on titanium or aluminium is comparable. In general, biocompatibility is determined by the chemical composition of the boundary layer. In the case of aluminium, the chemical composition of the present native oxide layer is identical to the ceramic Al_2_O_3_, which is already used as material for implants (Saini et al. 2015; Spriano et al. 2017). Aluminium and titanium are also established metals for printed circuit board manufacturing processes; thus, these metals are considered highly suited for their application as sensor materials of lab-on-a-chip devices (Mazzuferi et al. 2010). Concerning electrode design, different variants have been tested for ECIS applications. Two of these variants involve circular coplanar electrodes with a small working electrode and a large reference electrode (Zhang et al. 2017), and interdigitated electrodes (Wang et al. 2008). Interdigitated electrodes (IDE) were reported to be suited for assessing functional cell layers due to their large effective sensing area (Liu et al. 2014; Mamishev et al. 2004).

For our study, we fabricated IDEs with an electrode width of 50 μm and an inter-electrode distance of 50 μm using three different materials: gold, aluminium, and titanium. The behaviour of these sensors was tested by applying reference electrolytes (potassium chloride solutions) and by assessing the barrier functionality of two epithelial cell models: renal proximal tubular *LLC-PK1* cells, known to establish functional tight junctions (Gstraunthaler et al. 1985; Lechner et al. 1999; Litkouhi et al. 2007), and the cervix carcinoma cell line *HeLa*, which lacks the ability to establish a functional epithelial barrier (Capaldo et al. 2014).

## Materials and methods

### Sensor fabrication

Impedimetric sensors were fabricated by photolithographic means. Conductive metal layers of aluminium (thickness 100 nm) and titanium (thickness 80 nm) were deposited on transparent substrates (100 mm diameter, Pyrex®-wafers) by using an LLS EVO sputter coating system (Oerlikon Balzers, Balzers, LI). The electrode material (gold, thickness 100 nm) was deposited by high vacuum evaporation (Univex 500, 3SC Leybold, Cologne, DE). Metal-coated wafers were heated for dehydration (10 min; 150 °C), followed by spin-coating of an adhesion promoter (Ti-Prime, Microchemicals GmbH, Ulm, DE). The wafers were then baked at 120 °C for 120 s. After cooling to room temperature (RT), the prepared substrates were spin-coated with a photoresist (Photoresist AZ 1518; Merck, Darmstadt, DE) until a final layer thickness of 2.4 μm was achieved. The soft bake was performed on a hotplate at 100 °C for 80 s. After cooling down to RT, the wafer was exposed in a SUSS Mask-Aligner MA6 for 2.5 s (dose 30 mJcm-2, broadband illumination). The photomask was designed with an etching undercut of 2 μm. After exposure, the photoresist was developed in 2.38% tetramethyl-ammoniumhydroxide solution in H_2_O (MIF Developer AZ-726, Merck, Darmstadt, DE) for 50 s. Finally, the substrates were rinsed in deionized water and air-dried within a developer station (EVG 101, EV Group, St. Florian am Inn, AT). For the titanium sensors, the titanium coated wafers were etched in 1% HF solution for 60 s. For the aluminium sensors, an aluminium etchant (ANPE 80/5/5/10, Microchemicals GmbH, Ulm, DE) was utilized. The etch time for the 100 nm thick aluminium layer was 4 min. The gold coated substrates were etched in KI:I_2_ mixture (KI:I_2_:H_2_O = 4 g:1 g:40 ml) for 30 s. Finally, the photoresist was stripped in an acetone bath followed by an isopropanol - deionized water rinse.

### Measurement setup

Prior to impedance experiments, the wafers were sterilised by incubation in EtOH (70 vol-%) for 10 min followed by subsequent washing using sterile phosphate buffered saline. In order to provide separated active sensing areas on the glassy wafers, in-house fabricated and sterilised silicon (Elastosil M 4601 A/B, Wacker Chemie AG, Munich, DE) compartments were positioned on the wafers in alignment with the sensor structures. To ensure liquid tightness, the bottom sides of the silicone compartments were lubricated using multi-purpose silicone grease (OKS 1110 – Multi-Siliconfett, OKS, Maisach, DE). The active sensing areas (interdigitated electrodes) were connected to square contact pads and to a vector network analyser (Bode 100, Omicron electronics GmbH, Klaus, AT) in a balanced setup. Absolute impedance, real and imaginary part of impedance, as well as phase shifting were obtained by the data acquisition and analysis software Bode Analyzer Suite v2.43SR1 (Omicron electronics GmbH, Klaus, AT) at 401 logarithmically distributed frequencies ranging from 100 Hz to 40 MHz.

### Impedance of reference electrolytes

Potassium chloride (#P9541, Merck, Darmstadt, DE) was dissolved in bidest. Water at defined concentrations (0.01 M, 0.1 M, 1 M). For impedance measurement, each compartment was filled with 150 μl of the electrolyte. One measurement series consisted of six repeated measurements. Three series were obtained with electrolyte samples taken on two different days. Based on the measurements, sensor precision was determined in accordance with the procedures described by Hedderich and Sachs (2016). The first parameter of sensor precision assessed was the repeatability of all series. The second parameter was the reproducibility calculated by comparing the measurements between the two measurement days. Stability, the third parameter, described the precision of the measurement system over all series on independent measurement days. Finally, all three parameters (repeatability, reproducibility, and stability) were combined to determine the sensor’s uncertainty factor (Hedderich and Sachs 2016).

### Cell culture

Cell culture (*HeLa* and *LLC-PK1*) was prepared in accordance with the recommendations of the ATCC (American Type Culture Collection, http://www.lgcstandards-atcc.org/). Two cell lines were chosen as *in-vitro* models: *HeLa* (human epithelioid cervix carcinoma) and *LLC-PK1* (porcine normal renal proximal-tubular cell line). Both cell lines were maintained in Dulbecco’s Modified Essential Medium (DMEM; #BE12-707F; Lonza, Basel, CH) supplemented with 10% fetal bovine serum, penicillin, streptomycin, and 2 mM glutamine (#10378–016; Thermo Fisher Scientific, Waltham, US). For sub-culturing, nearly confluent cell monolayers were detached from the growth support through treatment with trypsin (#T4174; Merck, Darmstadt, DE). Each sensor well was seeded with 150 μl cell suspension at a cell density of 250 cells/m^2^. To avoid contamination during analyses, silicone compartments were covered by coverslips. Sensor setups were placed inside an incubator (37 °C, 5% CO2) to maintain physiological atmospheric conditions. Cell growth was documented by phase contrast light microscopy. Image acquisition was done using an inverted microscope (TE eclipse; Nikon, Tokyo, JP) at 100/200-fold magnification.

### Impedance data analysis

The impedance recordings of a cell culture medium without cells were used as a reference measurement. For the initial analysis of the impedance data, the absolute impedance was plotted against the measurement frequency. To calculate the relative impedance change, the impedance change was determined by subtracting the reference impedance spectrum for any given impedance spectrum. The relative impedance change was determined by dividing the impedance change by the reference impedance.

### Impedance data fitting

For analysis of the measured impedance spectra, the parameters of two different electrical models were numerically fitted to the normalized impedance spectra. The fitting process was executed with MATLAB (2018b, MathWorks, North Natick, US) applying a nonlinear least-square solver. Raw impedance data was multiplied by the active sensor area (Au: 0.1246 cm^2^; Al: 0.1246 cm^2^; Ti: 0.1245 cm^2^) and the number of gaps (n_Gap_ = 39). The resulting impedance data represent the average impedance between two adjacent electrode fingers normalized to the exposed area of the sensor.

The first model is based on the work by Robilliard et al. (2018) and consists of the ohmic resistances R_bulk_ (representing the combined resistance of the cell culture medium, electrode traces, and measurement wiring), R_cleft_ (resistance of the sub-cellular space underneath the cell layer), and R_cell-layer_ (resistance of the cell layer). The capacitive elements of the EEC were C_interface_ (interface capacitance) and C_cell-layer_ (capacitance of the cell layer) (see Fig. 4). The bulk resistance R_bulk_ was determined by calculating the mean value of the real impedance part in the high frequency range (10 MHz – 20 MHz). The parameter for the interface capacitance C_interface_ was determined by fitting the parameters of the constant phase element to the measured data in the low frequency range (Au / Al: 100 Hz – 185 Hz; Ti: 100 Hz – 157 Hz). The resulting equation for the total impedance of the model was used as basis for the optimization process. The parameters of the model were optimized to minimize the distance between the measured data and the theoretical data. For the mathematical fitting process, the absolute impedance at the frequencies 100 Hz, 125 Hz, 247 Hz, 501 Hz, 1.019 kHz, 2.007 kHz, 3.95 kHz, 8.03 kHz, 16.324 kHz, 32.131 kHz, 63.246 kHz were used. To avoid unbalanced fitting, the modelled and measured impedance were weighted to a serial circuit consisting of the interface capacitance and the bulk resistance.

The second model is based on the work by Giaever and Keese (1991), which describes the cell layer based on differential equations of the electric field. The parameters of the model are Z_c_ (measured impedance), Z_n_ (impedance of the cell-free state), Z_m_ (impedance of the cell membrane), α (constraint of current flow in the sub-cellular space), and R_b_ (resistance between the cells). The analysis of the parameters of the two models was performed separately for three sensing fields. The resulting graphs represent the mean and the standard deviation of the three sensing fields.

### Microscopic observation of cell growth

Cell growth of *LLC-PK1* and *HeLa* cells was observed and recorded by a confocal microscope (Eclipse TE300, Nikon, Tokyo, Japan) at 3 h, 24 h, 48 h, 72 h, 96 h, 120 h, 144 h, and 168 h. Images were acquired with the software NIS-Elements F 2.20 (Nikon, Tokyo, Japan).

### Statistics

Statistical data analysis was done by using the software MATLAB (R201bb, MathWorks, North Natick, US). The analysis of variance (ANOVA) and student-t test was applied to test for statistical significance. The significance level was set to 0.01.

## Results

### Measurements of the conductivity of defined electrolytes

**Fig. 1:**
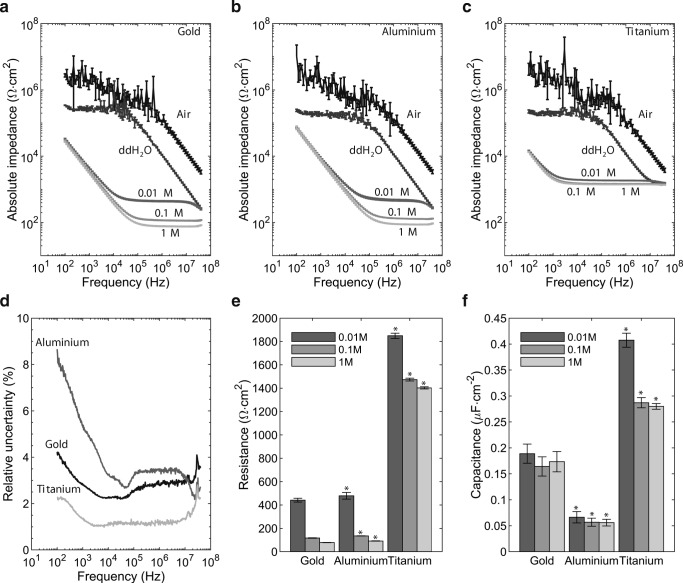
Characterization of the impedimetric sensors built of gold, aluminium, and titanium by using defined test agents. Air, distilled water and potassium chloride solutions (KCl) at 0.01 M, 0.1 M, and 1 M were used as test agents to compare gold, aluminium, and titanium sensors. Figure a, b, and c display the whole frequency spectra of absolute impedance (mean of 6 measurements; error bars show the standard deviation of the mean). Resistance and capacitance calculated from the spectra are shown in d (R_sensor_) and e (C_interface_) for each electrolyte and sensor material. Figure d displays the mean of the uncertainty in percent of the mean of the relevant measurements, combining the measurements of all three KCl concentrations at a single frequency for each sensor material. The means of six measurements are displayed, error bars represent the standard deviation from the mean. Statistically significant difference to the respective measurement on gold sensors at *p* < 0.01 is marked by an asterisk (*)

Impedance measurements on air with gold, aluminium, and titanium sensors showed a purely capacitive behaviour indicated by decreasing absolute impedance with increasing measurement frequency (see Fig. 1a, b, and c). Adding bidest water to the sensors caused an even downshift of absolute impedance in combination with an impedance plateau at lower frequencies (see Fig. 1a, b, and c). The impedance spectra of these two test substances (air and bidest water) were similar between the three sensor materials gold, aluminium, and titanium.

The impedance spectra of potassium chloride solutions of three different concentrations (0.01 M, 0.1 M, and 1 M) were characterized by two distinct frequency dependant regions, independently of the sensor materials (see Fig. 1a, b, and c). The first region (low-frequency range) was characterized by a continuous decrease of absolute impedance indicating a capacitive behaviour, whereas the second region was marked by a constant value of absolute impedance indicating a resistive behaviour. Impedance spectra of KCl electrolytes assessed by aluminium and gold electrodes exhibited comparable characteristics. Titanium sensors differed from gold and aluminium within the high-frequency region. Titanium showed an about 10-fold higher value of absolute impedance compared to gold (see Fig. 1c).

Determination of electrolyte resistance and electrolyte capacitance of the electrolyte were determined by fitting the parameters of a simple equivalent electric circuit (EEC) to the measured impedance spectra. The applied EEC consisted of two elements: a constant phase element (C_interface_) and an ohmic resistance (R_sensor_). As shown in Fig. 1e, the resistance was influenced by the ionic concentration and the electrode material. With increasing concentrations of potassium chloride, the values of the R_sensor_ decreased (see Fig. 1d). Aluminium sensors displayed equivalent R_sensor_ values as gold sensors. In contrast, the resistances measured by the titanium sensors were higher compared to those reported by gold sensors. The capacitance C_interface_ assessed by aluminium and gold electrodes was not influenced by the potassium chloride solutions (see Fig. 1e). However, the electrolyte capacitance on aluminium sensors was only half of the capacitance compared to gold. On the other hand, the capacitance determined by the titanium electrode was higher than the capacitance reported by gold sensors. Unlike gold and aluminium sensors, titanium sensors exhibited a higher capacitance of the 0.01 M KCl electrolyte than of the 0.1 M and 1 M KCl electrolyte solution.

### Precision of absolute impedance measurements

The precision of measurements was represented by the parameter uncertainty, which considers the parameters repeatability, reproducibility, and stability (Hedderich and Sachs 2016). The uncertainty parameter (shown relative to the measurement value) was frequency dependant for all three materials (Fig. 1f). In the low-frequency region, the uncertainty decreased with increasing frequency, whereas at higher frequencies the uncertainty was more stable. At low frequencies, aluminium sensors exhibited the highest uncertainty, followed by gold and finally titanium sensors. In the high-frequency range, titanium sensors displayed the lowest uncertainty in contrast to aluminium and gold sensors, which shared similar readings in that both indicated an uncertainty below 4%.

### Assessment of epithelial barrier properties by ECIS

**Fig. 2:**
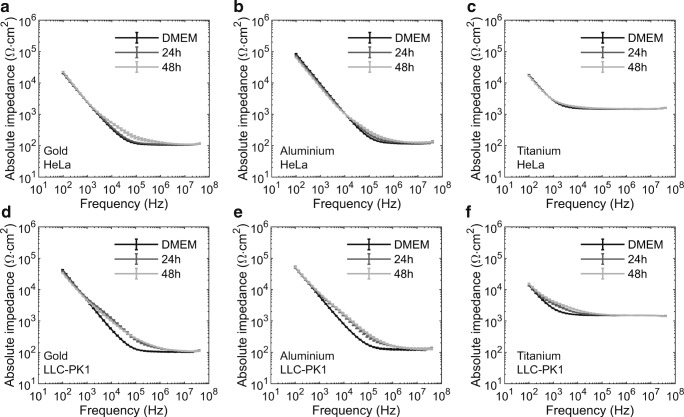
Measured impedance spectra of adherent cells. Functional (transporting) epithelial cells (LLC PK1; a, b, and c) and non-functional (transporting) cells (*HeLa*; d, e, and f) were cultured directly on the different impedimetric sensors made of gold (a, d), aluminium (b, e), or titanium (c, f). Absolute impedance was measured at 401 distinct frequencies logarithmically distributed in the range from 100 Hz to 40 MHz. Absolute impedance values were normalized to exposed sensor surface. Impedance spectra obtained at 24 h and 48 h after seeding of the cells are represented. In addition, the impedance spectra obtained with the cell culture medium alone are shown as reference. Each measurement point represents the mean of three independent measurements. For reasons of clarity, error bars, representing the standard deviation from the mean, are shown for every fifth data point only

After characterization of the sensors with defined electrolytes, *LLC-PK1* and *Hela* cells were seeded on the gold, aluminium, and titanium sensors to determine the performance of the different sensor materials when assessing cell behaviour. In comparison to cell culture medium alone, *LLC-PK1* and *HeLa* cell cultures altered the absolute impedance measurements in a frequency dependent manner. Within the initial 48 h after cell seeding, impedance alterations were detected in the low- and high-frequency regions for both cell types (Fig. 2).

**Fig. 3:**
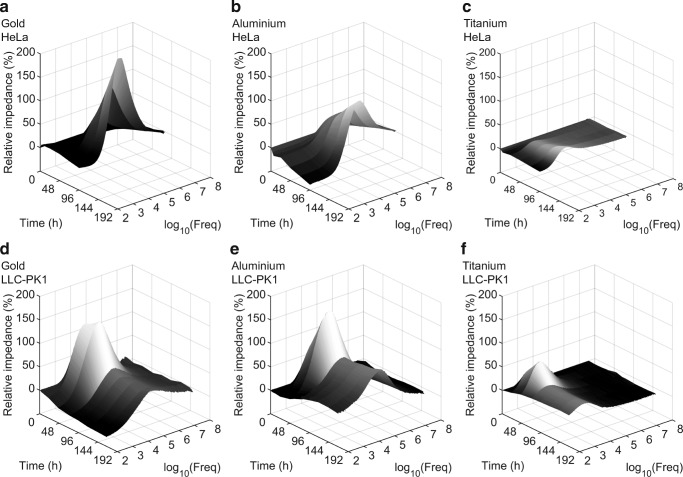
The relative changes of the impedance induced by cell activity is influenced by the measurement frequency. Relative changes of impedance are expressed as differences between the measurements with and without cells in percent of the measurement with cell culture medium alone. Spectra of relative impedance changes are shown for *HeLa* (a, b, c) and LLC-PK1 (d, e, f) cell cultures obtained between 3 and 96 h after seeding. Results from gold electrodes are shown in a and d, from aluminium in b and e, and from titanium in c and f

To further investigate the frequency-dependency of impedance alteration, the relative change of impedance was calculated for the two cell models and the three sensor materials (see Fig. 3). The most-sensitive region for the impedance change was identified at the separation frequency, which marked the transition from the resistive to the capacitive dominated region. In the case of *LLC-PK1* cells, the maximum relative change of absolute impedance at the separation frequency was reached between 24 h and 48 h. A comparison of the three sensor materials revealed that gold sensors (+132 ± 9%) achieved the highest change, followed by aluminium sensors (+130 ± 20%), and finally titanium sensors (+72 ± 11%). For *HeLa* cells, the maximum was reached 96 h after seeding. Here, too, gold sensors (+204 ± 29%) reported the highest change, followed by aluminium sensors (+95 ± 10%), and titanium sensors (+12 ± 2%).

### Equivalent electric circuit modelling of epithelial tissue

**Fig. 4:**
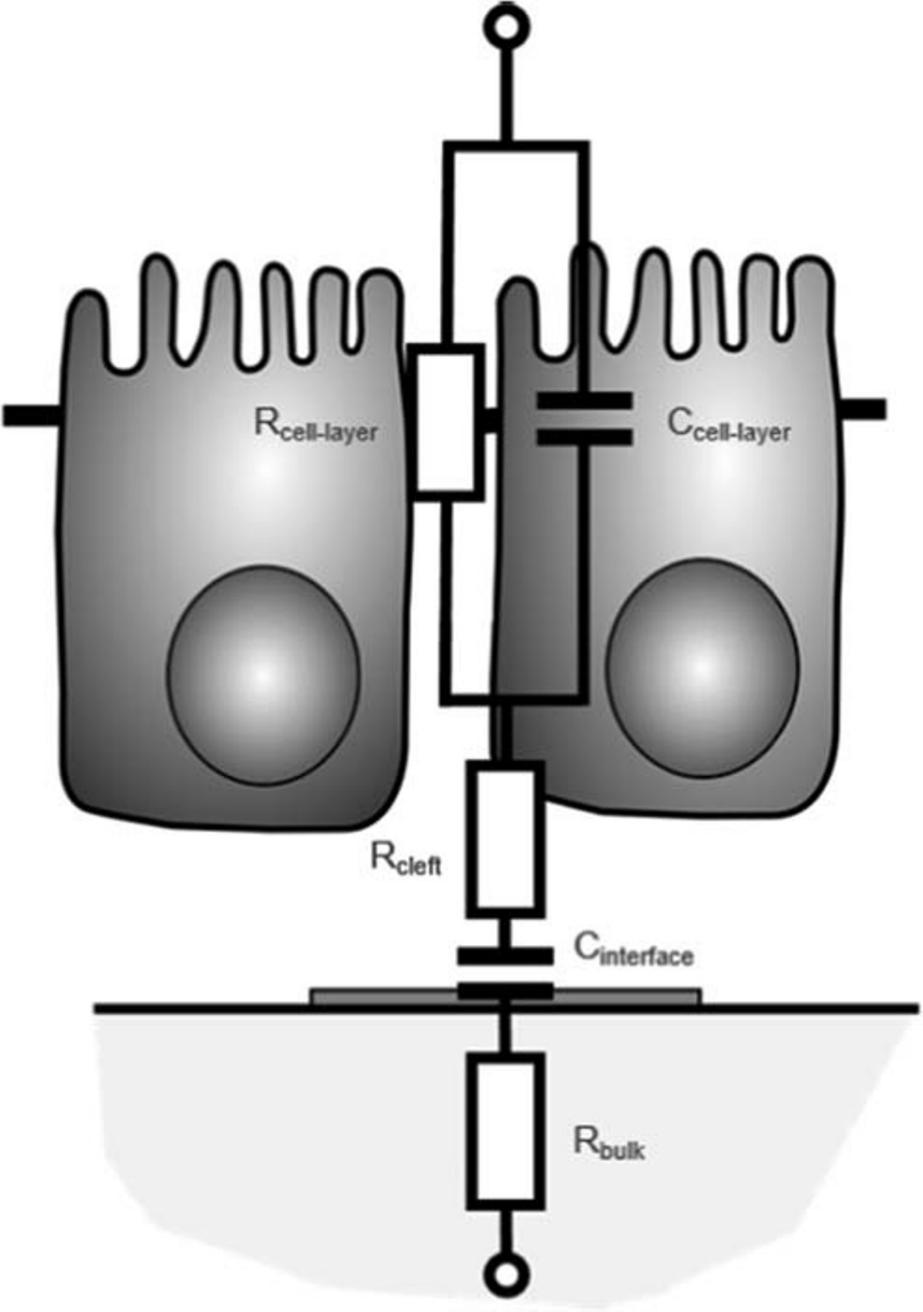
Equivalent electric circuit of the cell layer. To determine electrical parameters describing the cell layer, an equivalent electric circuit was applied according to Robilliard et al. (2018). The model consists of the ohmic resistances R_bulk_ (describing the resistance of the electrode traces and the resistance of the culture medium), R_cell-layer_ (describing the resistance of the cell layer), and R_cleft_ (describing the resistance of the sub-cellular cleft between the basolateral side of the cells and the electrode). The capacitances of the model are C_interface_ (capacitance of the interface including double-layer capacitance and material-specific boundary layer capacitance) and the cell layer capacitance C_cell-layer_

To analyse the behaviour of the cells in more detail, parameters of an equivalent electric circuit (EEC) were fitted to the measured impedance spectra. The EEC for modelling the cell layer is shown in Fig. 4 and is based on the work by Robilliard et al. (2018).

**Fig. 5:**
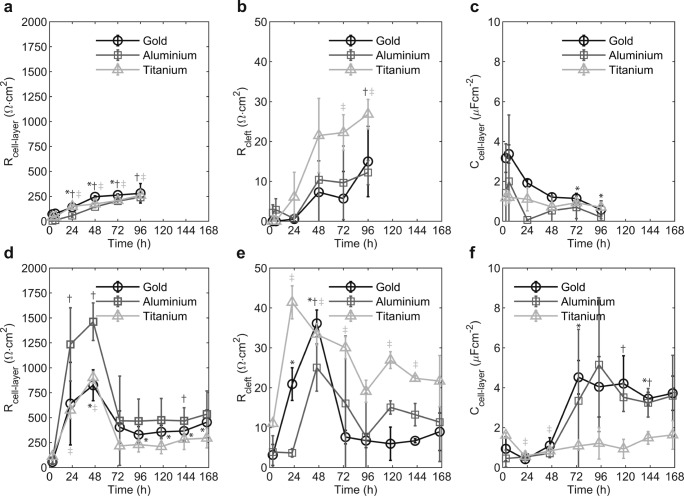
Results after fitting parameters of a model to measurement data. Parameters of an electrical model for epithelial cells were fitted to the measured and normalized impedance data. The parameter R_cell-layer_ displays the ohmic resistance of the cell layer and is shown in a (*HeLa*) and d (*LLC-PK1*) for gold, aluminium, and titanium sensors. The resistance of the sub-cellular space between the electrode and the basolateral side of the cell layer (R_cleft_) is shown in b (*HeLa*) and e (*LLC-PK1*) for gold, aluminium, and titanium sensors. Finally, the capacitance of the cell layer (C_cell-layer_) is displayed in c (*HeLa*) and f (*LLC-PK1*) for gold, aluminium, and titanium sensors. Statistically significant differences at p < 0.01 versus the measurements at 3 h and at the respective time point are marked by an asterisk (*) for gold sensors, by a dagger (†) for aluminium sensors, and by a double dagger (‡) for titanium sensors

For *HeLa* cells, the resistance R_cell-layer_ increased with cultivation time (see Fig. 5a). The assessed increase was independent of the sensor material. At the end of the experiment (96 h), R_cell-layer_ was 280 ± 98 Ωcm^2^ on gold sensors, 240 ± 41 Ωcm^2^ on aluminium sensors, and 260 ± 15 Ωcm^2^ on titanium sensors. The resistance R_cleft_ modelling the cell-substrate adhesion increased after 24 h to 48 h and reached a constant value (see Fig. 5b). At 96 h, R_cleft_ was 15 ± 9 Ωcm^2^ on gold electrodes, 12 ± 3 Ωcm^2^ at aluminium electrodes, and 27 ± 4 Ωcm^2^ on titanium electrodes. The capacitance of the *HeLa* cell layer after 96 h was 0.58 ± 0.4 μFcm^−2^ on gold sensors, 0.20 ± 0.07 μFcm^−2^ on aluminium sensors, and 0.71 ± 0.16 μFcm^−2^ on titanium sensors (see Fig. 5c).

In the case of *LLC-PK1* cells, the behaviour and the values for the parameters of the applied EEC were different in comparison to *HeLa* cells. The ohmic resistance of the cell layer (R_cell-layer_) exhibited an initial increase reaching a maximum between 24 h and 48 h (see Fig. 5d). After reaching its peak, the value decreased and finally stabilized. The measured peak differed between the sensor materials. The highest value for R_cell-layer_ was measured on aluminium sensors (1462 ± 188 Ωcm^2^), followed by titanium sensors (902 ± 58 Ωcm^2^), and finally gold sensors (825 ± 155 Ωcm^2^). The peak assessed on aluminium was statistically significantly higher in comparison with gold and titanium. Between 72 h and 168 h, the value for R_cell-layer_ stabilized at 383 ± 104 Ωcm^2^ for gold, at 483 ± 231 Ωcm^2^ for aluminium sensors, and at 247 ± 76 Ωcm^2^ for titanium sensors. The value of R_cell-layer_ on titanium sensors was statistically significantly lower than the values achieved with gold and aluminium sensors.

In the case of aluminium and gold sensors, the resistance of the cleft (R_cleft_) exhibited an initial increase peaking at 48 h – 72 h, followed by a decrease, and a final stabilization for the remaining cultivation time (see Fig. 5e). The resistance R_cleft_ in the stabilized phase was 7 ± 3 Ωcm^2^ for gold sensors and 13 ± 9 Ωcm^2^ for aluminium sensors. On titanium sensors, the value for R_cleft_ increased initially but in contrast to gold and aluminium sensors, the plateau remained until 72 h. After 72 h, the value decreased only slightly, stabilizing at 23 ± 4 Ωcm^2^. The capacitance of the epithelial cell layer increased between 48 h and 72 h, reaching a stable value of 4.0 ± 1.0 μFcm^−2^ on gold sensors and 3.8 ± 2.2 μFcm^−2^ on aluminium sensors (see Fig. 4f). On titanium sensors, the capacitance C_cell-layer_ remained at a constant value of 1.2 ± 0.5 μFcm^−2^ throughout the experiment. The value of C_cell-layer_ during the stable phase assessed on titanium sensors is statistically significantly lower than the value assessed using gold and aluminium sensors.

The impedance spectra were also analysed by applying the approach described by Giaver & Keese (Giaever and Keese 1991). The parameters of this model are R_b_ (barrier resistance), α (cell-substrate adhesion), and C_m_ (capacitance of the cell membrane) which were determined for each impedance data set. The barrier resistance R_b_ of the *HeLa* cell model reached a value of 205 ± 10 Ωcm^2^ on gold sensors, 139 ± 14 Ωcm^2^ on aluminium sensors, and 35 ± 31 Ωcm^2^ on titanium sensors after 96 h cultivation. The cell-substrate adhesion described by α was 26 ± 1 Ω^0,5^cm on gold sensors, 20 ± 9 Ω^0,5^cm on aluminium sensors, and 24 ± 1 Ω^0,5^cm on titanium sensors at 96 h (see Table 1).

**Table 1:** Cell layer parameters

Cell Model	HeLa (96 h)		LLC-PK1 (24 h–48 h)		LLC-PK1 (72 h–168 h)	
	*R*_*b*_(Ω*cm*^2^	*α*(Ω*cm*^05^	*R*_*b*_(Ω*cm*^2^	*α*(Ω*cm*^05^	*R*_*b*_(Ω*cm*^2^	*α*(Ω*cm*^05^
Gold	205±10	26±1	528±26	–	149±73	4±3
Aluminium	139±14	20±9	490±75	–	37±36	41±15
Titanium	35±31	24±1	204±49	–	7±9	4±4

The functional epithelial cell model (*LLC-PK1*) exhibited a different behaviour of the parameters R_b_ and α. The barrier resistance R_b_ increased initially reaching a peak between 24 h and 48 h. All three sensors materials detected this initial peak. The value of R_b_ at the peak was 528 ± 26 Ωcm^2^ on gold sensors, 490 ± 75 Ωcm^2^ on aluminium sensors, and 204 ± 49 Ωcm^2^ on titanium sensors. During the stabilization phase (72 h – 168 h), the value for R_b_ was 149 ± 73 Ωcm^2^ on gold sensors, 37 ± 36 Ωcm^2^ on aluminium sensors, and 7 ± 9 Ωcm^2^ on titanium sensors. The value of α exhibited a similar characteristic as R_b_, in that after an initial increase and reaching a peak, α dropped and stabilized throughout the remaining experimental time. In the stable phase, the value of α was 4 ± 3 Ω^0.5^cm on gold sensors, 41 ± 15 Ω^0.5^cm on aluminium sensors, and 4 ± 4 Ω^0.5^cm on titanium sensors (see Table 1).

**Fig. 6:**
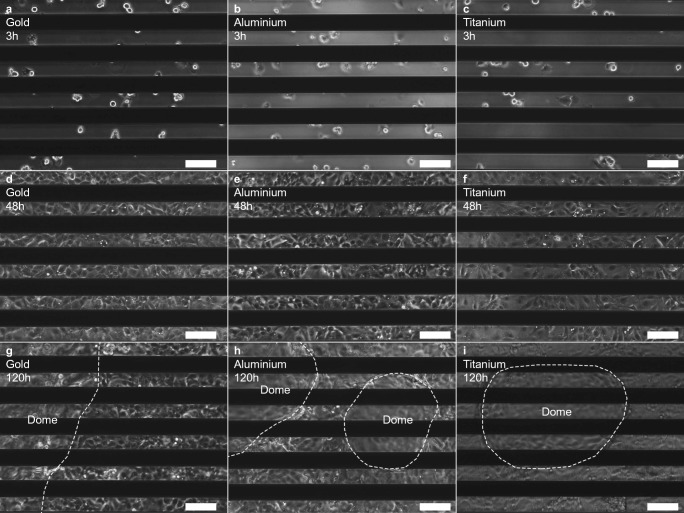
Functional epithelial cells on gold, aluminium, and titanium sensors. Functional epithelial cells (*LLC-PK1*) were seeded on interdigitated sensors made of gold (a, d, and g), aluminium (b, e, and h), and titanium (c, f, and i). Microscopical images were recorded 3 h after seeding (a, b, and c) representing the adhesion phase. Rounded and adhered cells are visible. The second image series were taken after 48 h cultivation marking the peak phase (d, e, and f). The cells reached confluency after 48 h of growth displayed by a closed cell layer. Finally, images were recorded after 120 h (g, h, and i) within the stable phase. During this phase, dome structures were visible which are known as differentiation markers indicating a transporting layer of *LLC-PK1* cells. Dome borders are marked with a white, dashed line. White bars within the images are 100 μm in length

The characteristic behaviour of the electrical parameters relative change of absolute impedance, R_cell-layer_, and R_b_ of the *LLC-PK1* model enabled a distinction between three growth phases: initial phase (indicated by an increase in the parameters), peak phase (indicated by reaching a peak of the parameters), and a stable phase (indicated by constant values of the parameters). Microscopic observation revealed that during the initial phase, the cells adhere to the substrate and proliferate (see Fig. 6a, b, and c). At the peak phase, a confluent cell layer was present (see Fig. 6 d, e, and f). During the stable phase, the presence of ‘dome’ structures was detected (see Fig. 6g, h, and i). Domes are known as differentiation markers of functional *LLC-PK1* cell layers indicating ongoing transepithelial transport processes.

## Discussion

In the current report, the applicability of aluminium and titanium as alternative sensor materials to gold for measuring barrier functionality of epithelial tissues *in vitro* was studied. At first, sensor materials were tested for assessing the impedance spectra of defined electrolyte solutions. Then, two cell models (human cervix carcinoma cells and porcine renal proximal tubular cells) were used to determine the measurement ability of the investigated sensor materials for assessing cells *in vitro*. For determination of epithelial barrier properties, the parameters of two electrical models were fitted to the measured impedance spectra.

The measured impedance spectra of air and bidest. Water with the three sensor materials were dominated by a capacitive region at high frequencies. The capacitance in this region describes the parasitic capacitance and represents the capacitances of the measurement setup. The vertical shift in impedance spectra between air and bidest. Water is attributed to the difference in relative permittivity of the medium on the sensors. The impedance spectra of air and bidest. Water of all three investigated materials was nearly identically. This region vanished when potassium chloride solutions were used as test liquid. For potassium chloride solutions, two dominant frequency-dependant regions were identified: a capacitive-dominated region at lower frequencies and a resistive-dominated region at higher frequencies. The capacitive region represents the capacitances, whereas the resistive region represents the ohmic resistances of the measurement system.

All three sensor materials were found to be suitable for assessing the solution resistance of potassium chloride electrolytes within the defined concentration range (0.01 M – 1 M). Aluminium sensors assessed a solution resistance comparable to gold sensors whereas titanium sensors exhibited elevated levels of the solution resistance. This observed increase is most probably due to the differences in electric conductivity between the sensor materials. The sensor traces from the sensing field to the landing pads for the measurement probes adds an additional ohmic resistance to the impedance spectra. The dimension of these traces is similar between the sensor materials; however, the electrical conductivity differs and is material-dependent. Consequently, the electrical conductivity of titanium is lower compared to gold and aluminium resulting in higher values for the ohmic resistance.

The differences in the interface capacitance between the sensor materials may be attributed to material specific and electrolyte specific differences. The interface capacitance includes the double-layer capacitance, which is influenced by the sensor material and the electrolyte (e.g. ion concentration). On the other hand, the interface capacitance also includes effects from the sensor material. In contrast to gold, aluminium and titanium sensors exhibit oxide passivation layers at the boundary layer. Thus, the oxide layer may also alter the interface capacitance. The interplay between double-layer capacitance and oxide-layer capacitance may cause the observed differences in the interface capacitance.

The influence of the electrode material on the uncertainty differed depending on the measurement frequency. In the low-frequency range, relative uncertainty of all three materials decreased and stabilized within the high-frequency range. All three materials exhibited a relative uncertainty below 4% within the high-frequency region. Thus, all three materials can be utilized to assess the conductivity of an electrolyte.

In order to determine the suitability of the different electrode materials for analysis of the epithelial barrier functionality, we assessed the ability of each material to determine cell layer specific parameters of two cell models: a transport competent (functional) epithelial model (*LLC-PK1*) and a transport incompetent (non-functional) epithelial model (*HeLa*). The spectra of absolute impedance as shown in Fig. 2 reveal the differences between the cell models. It shows that the impedance alterations in the case of the functional epithelial cell model (*LLC-PK1*) occurred in a broader frequency range than the impedance alterations induced by the non-functional cell model (*HeLa*). As reported in previous works, representation of relative impedance changes serves better to visualize frequency-dependent changes than spectra of absolute impedance (Daza et al. 2013). This type of data presentation enables the identification of the frequency range with the highest measurement sensitivity to cell activity, which occurs at the transition frequency separating the capacitive from the resistive dominated region.

For a detailed interpretation of the impedance data, the raw impedance spectra obtained from the cell cultures over time were analysed by fitting the parameters of two models to the assessed impedance spectra. The first model consists of ohmic resistances and capacitances, each element describing an electrical functionality of the cell model. The second model is based on the work by Giaever and Keese and represents a mathematical model of the cell layer. In the case of the non-functional cell model (*HeLa*), the fitting process based on the measured impedance spectra delivered comparable parameters for all three sensor materials. The resistance R_cell-layer_ (describing the tightness of the layer) increased with increasing cultivation time. The *HeLa* cells do not form tight epithelia and perform active transepithelial transport processes. Consequently, the parameter R_cell-layer_ does not reflect transport functionality; instead, cell proliferation induced sensor coverage. The resistance R_cleft_ describes the resistance of the space between the basolateral side of the cell layer and the electrode. For *HeLa* cells, R_cleft_ remains constant on aluminium and gold sensors. On titanium sensors, R_cleft_ increased with cultivation time. The increase may indicate a higher and over time increasing adhesion strength of *HeLa* cells on titanium sensors. The increase in R_cleft_ may be attributed to the excellent biocompatibility of titan which fosters strong adhesion between *HeLa* cells and the underlying titan electrodes (Saini et al. 2015).

The cell layer capacitance exhibited initial high values followed by a stabilization at around 1 μFcm^−2^, which corresponds with reported values for cell membrane capacitance in other studies (Bagnaninchi and Drummond 2011; Robilliard et al. 2018).

The functional epithelial cell model (*LLC-PK1*) exhibited a different behaviour of the parameters. The cell layer resistance R_cell-layer_ increased initially, reaching a peak, followed by a decline, and a final stabilization. This behaviour was recognized by all three sensor materials. The observed resistance profile was previously also described for *LLC-PK1* cell cultures using TEER measurements (Saladik et al. 1995). Regarding the initial TEER peak, a concomitant high degree of incorporation of radioactively labelled thymidine within the cells was detected during the first days of culture (Saladik et al. 1995). This indicates a high rate of de novo DNA synthesis resulting from cell proliferation matching our finding of an increase of the total protein content during the first days of *LLC-PK1* cell culture showing peak resistance values. It can be concluded that *LLC-PK1* cells still proliferate during this phase and that the increased proliferation causes an increase in resistance. This hypothesis is supported by the finding that active induction of proliferation by treatment of *LLC-PK1* with epidermal growth factor also increased the TEER (Saladik et al. 1995) and that the inhibition of proliferation by treatment with a specific inhibitor of the mitogen-activated kinases Erk1 and 2, key proteins involved in proliferative signalling pathways, not only blunts proliferation, but also the TEER increase (Lechner et al. 2007). In leaky epithelia like in *LLC-PK1* monolayers, TEER mainly reflects the ion permeability of the tight junctions. Incorporation of different transmembrane proteins of the claudin protein family within the tight junctions have been shown to determine the ion permeability of the epithelial tissue (Zihni et al. 2016). Claudin-2, a pore-forming claudin protein (Muto et al. 2010), was shown to be increasingly expressed among other claudins in human renal proximal tubular cells during the transition from a proliferative to a transporting phenotype (Aschauer et al. 2013). Proliferation was accompanied by the incorporation of tighter claudin proteins and a down-regulation of the pore-forming claudins (e.g. Claudin-2) within the tight junctional complex in other cell contexts as well (Balkovetz et al. 2004).

The values for R_cell-layer_ of matured *LLC-PK1* cells grown on gold and aluminium sensors were comparable to the values reported for the TEER by the study of Saladik et al. (Saladik et al. 1995). On titanium sensors, however, the value for R_cell-layer_ was significantly lower. The resistance R_cell-layer_ resembles the transport functionality of the epithelial tissue. Consequently, a reduced value may indicate a higher rate of transport activity. The ohmic resistance R_cleft_ resembles the adhesional strength of the cells on top of the electrode structures. The observed time pattern for R_cleft_ shows similarities with the time pattern of R_cell-layer_ – an initial increase, followed by a peak between 24 h and 48 h, and a final stabilization. As R_cleft_ resembles the adhesion between the cells and the electrode, the pattern may be caused by the partial lift-off of the cell layer due to dome-formation.

This R_cleft_ value reached a maximum on titanium sensors for both cell models. Given the possible higher transport activity combined with an increased cell adhesion, titanium may foster a highly bioactive growth environment compared to gold and aluminium. Several studies proved that titanium results in improved biocompatibility in the sense of growth rate and cell outgrowth compared to gold and aluminium (Niinomi 1999; Räisänen et al. 2000).

The capacitance C_cell-layer_ of the functional epithelial cell model increased on aluminium and gold sensors after 72 h and stabilized at an elevated level. Matured epithelial cells are characterized by an established brush border (Nielsen et al. 1998; Pfaller et al. 1990). A study with HEK-293 cells showed that an increase of microvilli caused an increase of the cell membrane capacitance (Zimmermann et al. 2008). The same effect may be the cause of the measured increase of the value C_cell-layer_. Cells cultivated on titanium electrodes did not cause an increase in C_cell-layer_. This might be attributed to the sensitivity of titanium sensors in terms of capacitive effects. As shown in Fig. 1e, the interface capacitance on titanium sensors is 2-times higher than on gold and aluminium sensors. This increased value for the interface capacitance might interfere biological induced alterations of capacitive parameters.

Finally, we applied a numerical cell model developed by Giaever and Keese for determining the barrier resistance R_b_, and the parameter α describing the current flow constraint within the subcellular cleft. The barrier resistance of the non-functional *HeLa* cell model increased with cultivation time. By contrast, the functional *LLC-PK1* cell model exhibited a peak of R_b_, followed by stabilization at lower values, indicating active transport processes. The highest values of R_b_ for the two cell models were achieved using gold sensors. The assessed value for R_b_ on gold sensors corresponds with the reported values of the transepithelial electrical resistance (TEER) of matured *LLC-PK1* cells (Lechner et al. 2007). On the other hand, aluminium and titanium sensors delivered significantly lower values for R_b_. The parameter α of the non-functional cell model was comparable between the three sensor materials. The functional cell model exhibited an increased value of α in the case of aluminium as sensor material. In contrast to aluminium, gold and titanium sensors exhibited a very low α value.

The model of Giaever and Keese applied was developed for gold electrodes and a different electrode setup (circular coplanar electrodes with a small sensing / working electrode and a large reference electrode) than the setup of the current work. As aluminium and titanium may add material specific impedance to the cell-growth induced impedance, the original model may have to be adapted to these conditions. Nevertheless, the resulting impedance spectra serve to determine the parameters R_b_ and α; thus, an adaption of the original model may improve the results of the expressive cell layer model.

## Conclusion

In conclusion, the investigated sensor materials, gold, aluminium, and titanium, were found to be suitable for assessing the functionality of epithelial tissue *in-vitro*. Aluminium sensors achieved similar readings as gold sensors, which may be attributed to the comparable electrical conductivities of the two materials. Titanium, however, showed to have an intrinsic elevated resistance, shifting the impedance readings to higher values. The reason for the shift may be titanium’s lower value for electrical conductivity compared to aluminium and gold.

The application of mathematical models to reveal cell-layer functionality proved appropriate for the three materials for the purpose of analysing ohmic resistances like cell-layer resistance and resistance of the sub-cellular cleft. The determination of capacitive parameters, such as cell-layer capacitance, delivered reasonable results for aluminium and gold, exhibiting an increase when a functional epithelial cell layer was present. However, titanium sensors exhibited a constant cell-layer capacitance despite the presence of a functional epithelial cell layer, as indicated by the occurrence of dome structures.

Visualization of the impedance as relative change of absolute impedance provided basic insight into sensor behaviour. Aluminium and gold sensors behaved similar in terms of sensibility to adherent and proliferating cells, whereas titanium sensors exhibited a moderate increase in relative impedance change, which was caused by the intrinsic elevated level of electrode trace resistance.

The functional epithelial cell line *LLC-PK1* altered the impedance in a broader frequency range compared to the non-function epithelial cell line *HeLa*. Whereas HeLa cells caused an incremental increase in impedance over cultivation time, the functional epithelial cell line LLC-PK1 caused an initial increase, peaking between 24 h and 48 h, followed by a decrease, and a final stabilization of the impedance at longer culture durations. The peak indicated a confluent cell layer where strong cell-cell contacts inhibit transport activity. The drop in impedance may be caused by maturation of the cell-cell contact complex (e.g. claudin expression), transforming the cell-layer to a transporting, functional epithelial cell-layer.

Aluminium and titanium proved to be suitable for assessing the functionality of an epithelial cell-layer. In combination with the application of mathematical models, the sensing system can reveal real-time data about the functionality of an epithelial cell-layer *in-vitro* non-invasively.
